# Perioperative opioids and survival outcomes in resectable head and neck cancer: A systematic review

**DOI:** 10.1002/cam4.6524

**Published:** 2023-09-14

**Authors:** Eric V. Mastrolonardo, Derek S. Mann, Harleen K. Sethi, Bo H. Yun, Elliott M. Sina, Maria Armache, Brooke Worster, Christopher E. Fundakowski, Leila J. Mady

**Affiliations:** ^1^ Department of Otolaryngology – Head and Neck Surgery Thomas Jefferson University Hospital Philadelphia Pennsylvania USA; ^2^ Department of Otolaryngology – Head and Neck Surgery Philadelphia College of Osteopathic Medicine Philadelphia Pennsylvania USA; ^3^ Department of Hospice and Palliative Care Thomas Jefferson University Hospital Philadelphia Pennsylvania USA; ^4^ Department of Otolaryngology – Head and Neck Surgery The Johns Hopkins School of Medicine Baltimore Maryland USA

**Keywords:** cancer management, clinical management, head and neck cancer, survival

## Abstract

**Background:**

Opioids are a mainstay in pain control for oncologic surgery. The objective of this systematic review is to evaluate the associations of perioperative opioid use with overall survival (OS) and disease‐free survival (DFS) in patients with resectable head and neck cancer (HNC).

**Methods:**

A systematic review of PubMed, SCOPUS, and CINAHL between 2000 and 2022 was conducted following the Preferred Reporting Items for Systematic Reviews and Meta‐Analyses guidelines. Studies investigating perioperative opioid use for patients with HNC undergoing surgical resection and its association with OS and DFS were included.

**Results:**

Three thousand three hundred seventy‐eight studies met initial inclusion criteria, and three studies representing 562 patients (intraoperative opioids, *n* = 463; postoperative opioids, *n* = 99) met final exclusion criteria. One study identified that high intraoperative opioid requirement in oral cancer surgery was associated with decreased OS (HR = 1.77, 95% CI 0.995–3.149) but was not an independent predictor of decreased DFS. Another study found that increased intraoperative opioid requirements in treating laryngeal cancer was demonstrated to have a weak but statistically significant inverse relationship with DFS (HR = 1.001, *p* = 0.02) and OS (HR = 1.001, *p* = 0.02). The last study identified that patients with chronic opioid after resection of oral cavity cancer had decreased DFS (HR = 2.7, 95% CI 1.1–6.6) compared to those who were not chronically using opioids postoperatively.

**Conclusion:**

An association may exist between perioperative opioid use and OS and DFS in patients with resectable HNC. Additional investigation is required to further delineate this relationship and promote appropriate stewardship of opioid use with adjunctive nonopioid analgesic regimens.

## 1 INTRODUCTION

Opioids are a mainstay in perioperative pain control for oncologic surgery. Concurrently, our country faces a public health crisis related to chronic opioid dependency and a significant increase in opioid overdose‐related deaths. Prescription of opioids has been rapidly increasing since the 1990s with the 2020 Center for Disease Control (CDC) reporting 43 opioid prescriptions per 100 people with a total of 142,816,781 prescriptions in 2020 alone; in 3.6% of counties, enough opioid prescriptions were dispensed for every person to have an opioid prescription.^1^

These alarming rates of opioid abuse are also prevalent in head and neck oncologic care. Postoperative opioid prescriptions in otolaryngology play a major role in developing opioid use disorder with reports demonstrating up to 10%–18% of previously opioid‐naïve patients developing chronic (persistent use 90 days after surgery) dependence after postoperative prescriptions.^2^, ^3^, ^4^ Chronic postoperative opioid use has been reported as high as 41% in oral cavity cancer and more than 50% in oropharyngeal squamous cell carcinoma.^5^, ^6^, ^7^

Studies analyzing opioids and cancer‐related outcomes are inconclusive. Mice models suggest opioids may promote pro‐tumor activity secondary to immunosuppression, migration of tumor cells, increased vascular endothelial factors receptors, and angiogenesis, but also, suggest anti‐tumor activity via apoptosis and phagocytosis.^7^ Clinical studies in oncologic care are also conflicting. In breast cancer, studies regarding opioids' link to cancer recurrence and survival are controversial at best with a multitude of studies either suggesting or refuting a connection between opioid use and patient survival and tumor recurrence rates.^8^

Opioid use and long‐term survival outcomes for head and neck cancer (HNC) surgery are not well reported in the literature. To address this knowledge gap, we performed the first systematic review to investigate the current state of the literature analyzing perioperative opioid use with oncologic survival outcomes in patients undergoing surgery for HNC.

## 2 METHODS

This systematic review followed the Preferred Reporting Items for Systematic Reviews and Meta‐Analyses (PRISMA) reporting guideline. Two authors (E.V.M., D.S.M.) searched three databases, PubMed, SCOPUS, and CINAHL, without restriction for study type, including retrospective and prospective cohort studies, cross‐sectional studies, and randomized clinical trials in English, published between 2000 and 2022, using the MeSH terms “head and neck cancer,” “surgical resection,” and “opioids.” For databases that did not use MeSH keywords, we added variants of the phrases “perioperative opioids”, “neoplasm” of all major head and neck subsites, and “surgical resection.”

Five authors (D.S.M., H.K.S., E.V.M., B.H.Y., E.M.S.) independently screened abstracts and titles followed by full texts to check the eligibility for inclusion. Article screening was completed using the online platform Covidence.

### 2.1 Inclusion and exclusion criteria

Studies were selected based on the following PICOS framework as follows:

*Population*: Adult patients with HNC.
*Intervention1*: Operative resection for HNC.
*Intervention2*: Perioperative opioid use near time of surgery.
*Comparator*: No perioperative opioid use (when possible).
*Outcomes*: Oncologic survival outcomes, including but not limited to overall survival (OS), disease‐free survival (DFS), and recurrence‐free survival (RFS).
*Study design*: Randomized and nonrandomized studies with an *n* > 1.

All studies that did not fit the population, intervention, and outcomes described below were excluded. The perioperative period was defined from 30 days preoperatively to 90 days postoperatively. Notably, esophageal cancer was excluded from our criteria for HNC. Database search queries were constructed using this framework and were optimized for each specific database. Search queries can be found in full in Appendix S1.

### 2.2 Data extraction

Two investigators (E.V.M., D.S.M.) independently screened the articles and extracted data from the included studies using a standard data extraction form. For each study, the following data were collected: study characteristics (first author, title, publication year, country, funding source, study design), study objectives, intervention details (e.g., intraoperative vs postoperative opioids), patient population (sample size, age and sex distribution), cancer treatment (cancer subsite, cancer stage, overall treatment including inclusion of chemotherapy or radiation), cancer pathology (pathologic stage, margin status, perineural extension, extracapsular extension), perioperative opioid characteristics (e.g., post‐discharge daily opioid dose, total post‐discharge opioids dispensed, fentanyl equivalents), and oncologic outcomes (OS, DFS/RFS, recurrence, persistence).

### 2.3 Outcomes

Coprimary outcomes included DFSand OS.

### 2.4 Risk‐of‐bias assessment

As all studies included in final analysis were retrospective cohort studies, risk of bias was assessed independently by investigators (E.V.M., D.S.M.) using the NIH Quality Assessment Tool for Observational Cohort and Cross‐Sectional Studies. The overall risk of bias was denoted as low concern, some concerns, or high risk based on the majority opinion. A high‐quality study was defined to have low risk of bias in all the assessed domains. The reviewers were not blinded to study details.

## 3 RESULTS

### 3.1 Study selection

**FIGURE 1:**
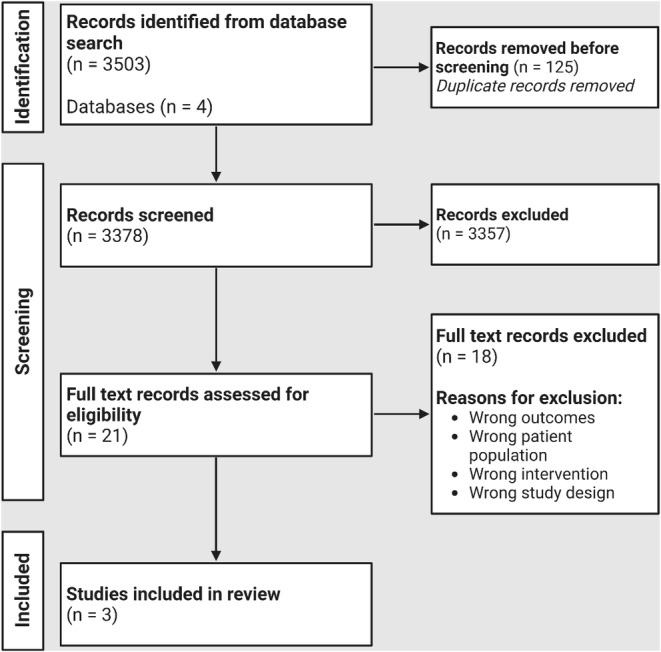
Preferred Reporting Items for Systematic Reviews and Meta‐Analyses flow diagram of studies in the systematic review.

The systematic query yielded 3503 studies in total across all databases, of which 3378 studies remained after deduplication for screening. Twenty‐one studies were then assessed for eligibility over full‐text review with three studies that remained from analysis. A PRISMA flow diagram demonstrating search results and study selection can be found in Figure 1.

Notable studies that were ultimately excluded include:

*Wrong patient population*: Oh et al.^9^ investigated high‐dose postoperative opioids in long‐term oncologic surgery outcomes, but it was in esophageal cancer, which was excluded from our definition of HNC.
*Wrong outcomes*: Saraswathula et al.,^4^ Hinther et al.,^10^ Lee et al.,^11^ and Cata et al.^12^ investigated persistent/chronic postoperative opioid use in HNC patients but did not investigate OS or DFS.
*Wrong intervention*: Zhang et al.^13^ investigated long‐term oncologic survival outcomes, but the associations were made with levels of mu‐opioid receptor (MOR) expression instead of opioid use.

### 3.2 Patient and study characteristics

All studies, which were retrospective in nature, were performed in the United States (*n* = 3%, 100%). Out of all participants (*n* = 463), the patients in Pang et al.^5^ (*n* = 99%, 18%) received postoperative opioids, while the patients in Patino et al.^14^ and Cata et al.,^15^ (*n* = 463%, 72%) all received intraoperative opioids. Disease subsites examined in included studies were oral cavity (*n* = 2%, 67%) and larynx (*n* = 1%, 33%). Study characteristics are shown in Table 1. All three studies were published between 2015 and 2017; although recent, no other studies that addressed our question have been published in the last 5 years.

**TABLE 1:** Characteristics of included studies.

Author, date	Country, study design	Details of investigated intervention	Cancer subsite(s)	Number of patients	Oncologic survival outcomes/associations
Patino et al. (2017)^14^	USA, retrospective cohort	Intraoperative opioids	Oral cavity	268	Intraoperative opioids on OS: HR = 1.77, *p* = 0.05 Intraoperative opioids on DFS: HR = 1.27, *p* = 0.26
Pang et al. (2017)^5^	USA, retrospective cohort	Postoperative opioid use	Oral cavity	99	Chronic postoperative opioid use on DFS: HR = 2.6 (95% CI: 1.1–6.6)
Cata et al. (2015)^15^	USA, retrospective cohort	Intraoperative opioids	Larynx	195	Intraoperative opioids on OS: HR = 1.001, *p* = 0.02 Intraoperative opioids on DFS: HR = 1.001, *p* = 0.02

Abbreviations: DFS, disease‐free survival; HR, hazard ratio; OS, overall survival.

Patino et al.^14^ investigated and identified the relationship between intraoperative opioid requirements and survival outcomes in a retrospective cohort of 268 patients undergoing surgical resection for oral cavity cancer. The authors reported that high intraoperative opioid requirement in oral cancer surgery was associated with decreased OS (HR = 1.77, *p* = 0.05) on multivariate analysis but was not an independent predictor of decreased DFS (HR 1.27, *p* = 0.26).^14^

Cata et al.^15^ investigated the relationship of intraoperative opioid requirements and survival outcomes in a retrospective cohort of 195 patients who underwent surgical resection for laryngeal cancer. The authors found that increased opioid requirements were demonstrated to have a weak but statistically significant inverse relationship with DFS (HR = 1.001, *p* = 0.02) and OS (HR = 1.001, *p* = 0.02).

Pang et al.^5^ investigated a retrospective cohort of 99 patients for associations of chronic postoperative opioid use with oncologic survival. Chronic postoperative opioid was defined as receiving multiple opioid prescriptions more than 90 days after surgery. On multivariate survival analysis, patients with chronic postoperative use had decreased DFS (HR = 2.7, 95% CI = 1.1–6.6) compared to those who were not chronically using opioids postoperatively.^5^ However, the analogous OS finding was not reported as the covariate was not retained for the corresponding survival function on multivariate analysis.^5^

### 3.3 Study quality analysis

Quality analysis of all three studies included was performed using the NIH Quality Assessment Tool for Observational Cohort and Cross‐Sectional Studies. The results of this assessment are included in Table S1.

## 4 DISCUSSION

We present the first systematic review to examine the association between perioperative opioids' use on overall and disease‐free survival in resectable HNC. Out of 3378 studies screened, only three studies met inclusion criteria, indicating a paucity of literature investigating this topic. Each study analyzed a different aspect regarding the association of opioid usage with survival in resectable HNC: intraoperative opioid requirement in oral cavity cancer; chronic postoperative opioid use in oral cavity cancer; and intraoperative opioid usage in laryngeal cancer.^5^, ^14^, ^15^ Interestingly, all three studies identified an association between opioid usage and worse survival outcomes.

The potential role of opioid usage in OS and DFS in oncologic survival outcomes is complex. All three studies in this systematic review suggest activation of MOR as a possible explanation due to its involvement in a plethora of tumorigenic pathways as demonstrated in preclinical studies across various cancer types.^5^, ^14^, ^15^

Opioids are divided into three categories: naturally occurring (morphine, codeine); semisynthetic (heroin, hydrocodone, hydromorphone, oxycodone, and buprenorphine); and synthetic (fentanyl, methadone, and tramadol).^16^ Most clinically relevant opioids are agonists of the MOR within the central and peripheral nervous systems.^16^ The major mechanism of action involves indirect activation of descending inhibitory neurons and a decrease in the transmission of nociception from the periphery to the thalamus.^16^ The *OPRM1* gene encodes for the MOR and has been reported to influence the response and dose needed to achieve pain relief.^16^, ^17^ A study by Viet et al.^18^ induced opioid tolerance in mice inoculated with oral SCC and observed *OPRM1* methylation and silenced mRNA expression. This resulted in the downregulation of MORs in the dorsal root ganglion of neurons and, thus, higher doses of opioid required to achieve pain relief in HNC mice models.^16^

In lung cancer in human models, MOR has been reported to be upregulated in non‐small cell lung cancer.^19^ In xenograft models, upregulated expression of MOR is implicated in increased primary tumor growth and metastases.^20^ Opioids may also induce cell proliferation and trigger epithelial‐mesenchymal transformation in lung cancer, and activation of MOR is also suggested to activate EGFR signaling pathways.^21^, ^22^ In breast cancer, MOR is associated with pro‐tumor activity via various pathways including interactions with glycogen synthase kinase 3, a multifunctional serine/threonine protein kinase whose aberrant expression contributes to progression of various disease processes including cancer.^23^, ^24^

Similar findings have been proposed and demonstrated in HNC. In vivo experiments discovered that treating various head and neck squamous cell carcinoma (HNSCC) cell lines with the highly selected MOR agonist DAMGO significantly increased cell proliferation, colony formation, invasion and migration, and promoted tumor growth.^25^ In studying 64 specimens from 32 matched patients with laryngeal carcinoma (supraglottic and glottic), MOR staining intensity was significantly increased in laryngeal carcinoma compared to the adjacent normal tissue.^26^

Two studies in this systematic review studying intraoperative opioid usage also propose a simpler, possibly more clinically relevant explanation for opioids' association with poor oncologic survival outcomes.^14^, ^15^ Both sets of investigators propose that patients with advanced disease, who typically have poor survival outcomes, undergo longer surgeries and therefore receive larger doses of opioids intraoperatively. As such, the observed association may be confounded by burden of disease (Table 2).^14^, ^15^ While the two explanations for opioid's association are not mutually exclusive, this theory remains a viable explanation that requires further investigation. It is important to note, however, that this theory does not explain the decreased disease‐free survival observed in one of the included studies.^5^

**TABLE 2:** Proposed mechanisms for association between opioid usage and decreased survival outcomes.

Author, date	Proposed mechanisms
Patino et al. (2017)^14^	1. Patients with advanced disease (and worse outcomes) ≥ longer operative times and more invasive surgeries ≥ increased opioid usage 2. Opioids induce tumor growth via induction of angiogenesis, immunosuppression, and mutagenic alterations
Pang et al. (2017)^5^	No proposed mechanisms
Cata et al. (2015)^15^	1. Opioids induce tumor growth via immune suppression, cell proliferation, and triggering epithelial‐mesenchymal transformation

Outside of the head and neck, the clinical data of opioids and oncologic survival outcomes are inconclusive at best. Retrospective review of 1111 radical prostatectomies for prostate cancer found that intraoperative sufentanil use was predictive of decreased disease‐free survival in patients undergoing radical prostatectomy in both univariate and multivariate analyses. Furthermore, retrospective review of 113 patients with metastatic prostate cancer found that increased MOR expression and greater opioid requirement were associated with decreased PFS and OS likely due to increased MOR‐induced signaling upon opioid exposure.^27^ Outside of prostate cancer, retrospective analysis of 99 patients with non‐small cell lung cancer demonstrated an association between increased doses of opioids with higher recurrence rates of NSCLC within 5 years.^28^

However, a multitude of literature suggests no association between opioids and worse oncologic survival outcomes. In triple‐negative breast cancer, retrospective analysis of 1143 patients found that intraoperative opioids were associated with favorable reduced recurrence‐free survival but not in overall survival.^29^ The investigators performed bulk RNA‐sequence analysis of opioid receptors and found that intraoperative opioid use was associated with upregulation of anti‐tumor receptors and downregulation of pro‐tumor receptors. Numerous other studies have demonstrated similar findings in both triple‐negative breast cancer and other cancer types.^30^, ^31^

It is important to note that other factors (metastatic burden, lymph node involvement, T stage, subsite of cancer, and medical comorbidities) play a significantly more important role in oncologic survival outcomes than do opioids.^32^ While this does not diminish the need for further investigation into the clinical relevance of opioids and HNC outcomes, it should be emphasized that opioid usage should not drive clinical decision‐making while current data are not backed by randomized controlled trials.

Socioeconomic status (SES), including low income and low education, predicts poor overall survival in HNC patients.^33^ Currently, there is no formal consensus on how to integrate SES into perioperative pain management for HNC patients. An association has also been established between long‐term opioid therapy and low SES in cancer survivors when low income, tobacco use, and unemployment are considered.^34^ Those associations coupled with the knowledge that opioid prescribing practices are higher in lower‐income cancer survivors highlights a challenge for HNC teams navigating this known risk factor.^35^

There are several noteworthy strengths, albeit acknowledged limitations, in this investigation. This is the first systematic review investigating the relationship between opioids and OS and DFS in resectable HNC. Furthermore, this review investigates an array of settings: perioperative (intra‐ and postoperative) and different subsites in the head and neck (larynx/hypopharynx and oral cavity). Nevertheless, this study also has several limitations. There is a paucity of studies investigating this topic with only three included of an initial 3378 studies screened, and the included studies demonstrated divergence in study design, methodology, and outcomes measured, thereby precluding the ability to perform a meta‐analysis. Furthermore, only retrospective studies were included in final analysis, which only allowed us to comment on reported associations rather than causative relationships between perioperative opioids and oncologic survival. No randomized clinical trials were identified in the systematic review. Given the limited number of studies that satisfied screening criteria, we were unable to conduct a meta‐analysis.

## 5 CONCLUSIONS

This systematic review is the first to examine the present literature investigating perioperative opioid use and its association with OS and DFS in HNC patients; results suggest an association may exist. Few retrospective studies have investigated this relationship, and these findings are critical in guiding prescribing practices in a population susceptible to opioid misuse. Additional investigation is required to further delineate this relationship and promote appropriate stewardship of opioid use with adjunctive nonopioid analgesic regimens.

## AUTHOR CONTRIBUTIONS

**Eric V. Mastrolonardo:** Conceptualization (equal); data curation (equal); formal analysis (equal); funding acquisition (equal); investigation (equal); methodology (equal); project administration (equal); resources (equal); software (equal); supervision (equal); validation (equal); visualization (equal); writing – original draft (equal); writing – review and editing (equal). **Derek S. Mann:** Conceptualization (equal); data curation (equal); formal analysis (equal); funding acquisition (equal); investigation (equal); methodology (equal); project administration (equal); resources (equal); software (equal); supervision (equal); validation (equal); visualization (equal); writing – original draft (equal); writing – review and editing (equal). **Harleen K. Sethi:** Data curation (equal); formal analysis (equal); investigation (equal); methodology (equal); resources (equal); writing – original draft (equal); writing – review and editing (equal). **Bo H. Yun:** Data curation (equal); investigation (equal); validation (equal); writing – review and editing (equal). **Elliott M. Sina:** Data curation (equal); investigation (equal); validation (equal); writing – review and editing (equal). **Maria Armache:** Data curation (equal); formal analysis (equal); writing – review and editing (equal). **Brooke Worster:** Investigation (equal); resources (equal); writing – review and editing (equal). **Christopher E. Fundakowski:** Investigation (equal); resources (equal); writing – review and editing (equal). **Leila J. Mady:** Conceptualization (equal); investigation (equal); methodology (equal); project administration (equal); resources (equal); supervision (equal); validation (equal); visualization (equal); writing – review and editing (equal).

## Supporting information


Appendix S1.

Table S1.

## Data Availability

Data available in article supplementary materials.
