# Supplementary material for: Perioperative opioids and survival outcomes in resectable head and neck cancer: A systematic review
Source: Cancer Med. 2023 Sep 14;12(18):18882–8. doi: 10.1002/cam4.6524 (PMC10557889; doi:10.1002/cam4.6524)
Supplement: Supplementary file 1 — Appendix S1. [file CAM4-12-18882-s002.docx]

**eAppendix.** Literature Search Strategy

PubMed

Search Executed: 10/26/2022

Number of Results: 3,039

Full Search Strategy:

("Head and Neck Neoplasms"[MeSH] OR "head and neck" OR "Skull Neoplasms"[Mesh])

AND

(Analgesics, Opioid [Pharmacological Action] OR "Analgesics, Opioid"[Mesh] OR "Narcotics"[Mesh] OR "Pain, Postoperative"[Mesh] OR "Buprenorphine"[Mesh] OR "Fentanyl"[Mesh] OR "Methadone"[Mesh] OR "Morphine"[Mesh] OR "Oxycodone"[Mesh] OR "Hydromorphone"[Mesh] OR "Postoperative Care"[Mesh] OR "opioid")

AND

("Otorhinolaryngologic Surgical Procedures"[Mesh] OR "Free Tissue Flaps"[Mesh] OR "Oral Surgical Procedures"[Mesh] OR 'resection' OR "Stomatognathic Diseases/surgery"[MeSH] OR "Head and Neck Neoplasms/surgery"[MeSH] OR "Skull Neoplasms/surgery"[Mesh])

CINAHL - EBSCOhost

Search Executed: 10/26/2022

Number of Results: 246

Full Search Strategy:

((MH "Head and Neck Neoplasms+") OR (MH "Skull Neoplasms+") OR TX (esophag* OR facial OR eyelid OR nose OR nasal OR paranasal OR lip OR ear OR auricular OR mouth OR jaw OR mandibular OR maxillary OR oral OR orbital OR "salivary gland" OR "sublingual gland" OR "submandibular gland" OR palatal OR gingival OR tongue OR tonsil OR tonsillar OR laryn* OR pharyn* OR otorhinolaryng* OR parathyroid OR thyroid OR trachea* OR "head and neck" OR nasopharyn* OR oropharyn* OR hypopharyn* OR oesophag* OR palate OR skull OR parotid OR ent OR orl OR "upper aerodigestive" OR "upper aero digestive" OR uadt ) N3 (neoplas* OR carcin* OR malignan* OR tumor* OR tumour* OR cancer* OR adenoma* OR "squamous cell carcinoma*") OR TX (hnc or scchn or hnscc or opscc))

AND

((MH ""Analgesics, Opioid+"") OR ""opioid*"" OR ""opiate*"" OR (MH ""Narcotics+"") OR ""narcotic*"" OR ((post-operat* OR postoperat* OR post-surg* OR postsurg* ) N3 (opioids OR 'narcotic' OR 'narcotics' OR 'opioid')) OR (alfentanil OR alphaprodine OR beta-casomorphin$ OR buprenorphine OR carfentanil OR codeine OR deltorphin OR dextromethorphan OR dezocine OR dihydrocodeine OR dihydromorphine OR enkephalin$ OR ethylketocyclazocine OR ethylmorphine OR etorphine OR fentanyl OR heroin OR hydrocodone OR hydromorphone OR ketobemidone OR levorphanol OR lofentanil OR meperidine OR meptazinol OR methadone OR methadyl acetate OR morphine OR nalbuphine OR opium OR oxycodone OR oxymorphone OR pentazocine OR phenazocine OR phenoperidine OR pirinitramide OR promedol OR propoxyphene OR remifentanil OR sufentanil OR tilidine OR tapentadol))

AND

((MH "Surgery, Otorhinolaryngologic+") OR (MH "Surgical Flaps+") OR resect* OR surg*)

SCOPUS - Elsevier

Search Executed: 10/26/2022

Number of Results: 331

Full Search Strategy:

(TITLE-ABS-KEY ((esophag* OR facial OR eyelid OR nose OR nasal OR paranasal OR lip OR ear OR auricular OR mouth OR jaw OR mandibular OR maxillary OR oral OR orbital OR "salivary gland" OR "sublingual gland" OR "submandibular gland" OR palatal OR gingival OR tongue OR tonsil OR tonsillar OR laryn* OR pharyn* OR otorhinolaryng* OR parathyroid OR thyroid OR trachea* OR "head and neck" OR nasopharyn* OR ORopharyn* OR hypopharyn* OR oesophag* OR palate OR skull OR parotid OR ent OR ORl OR "upper aerodigestive" OR "upper aero digestive" OR uadt ) W/3 (neoplas* OR carcin* OR malignan* OR tumOR* OR tumour* OR cancer* OR adenoma* OR "squamous cell carcinoma*")) OR TITLE-ABS-KEY (hnc OR scchn OR hnscc OR opscc))

AND

(TITLE-ABS-KEY (( post-operat* OR postoperat* OR post-surg* OR postsurg* ) W/3 (opioids OR 'narcotic' OR 'narcotics' OR 'opioid')) OR TITLE-ABS-KEY(buprenorphrine OR oxycodone OR methadone OR morphine OR fentanyl OR hydromorphone))

AND

(TITLE-ABS-KEY ("surgical resection" OR resect* OR excision OR "tissue flap" OR "free flap"))
