# Supplementary material for: Perioperative opioids and survival outcomes in resectable head and neck cancer: A systematic review
Source: Cancer Med. 2023 Sep 14;12(18):18882–8. doi: 10.1002/cam4.6524 (PMC10557889; doi:10.1002/cam4.6524)
Supplement: Supplementary file 2 — Table S1. [file CAM4-12-18882-s001.docx]

**eTable 1. Risk of Bias Assessment Consensus of Included Studies, NIH Quality Assessment Tool for Observational Cohort and Cross-Sectional Studies**

|  | Was the research objective clearly stated? | Was the study population clearly defined? | Was the participation rate of eligible persons at least 50%? | Were all subjects selected or recruited from the same populations? Were prespecified inclusion and exclusion criteria for being in the study and applied uniformly to all participants? | Was a sample size justification or power description provided? | Were the exposure(s) of interest measured prior to the outcome(s) being measured? | Was the timeframe sufficient so that one could reasonably expect to see an association between exposure and outcome if it existed? | Did the study examine different levels of the exposure as related to the outcome? | Were the exposure measures clearly defined, valid, reliable, and implemented consistently across all study participants? | Was the exposure(s) assessed more than once over time? | Were the outcome measures clearly defined, valid, reliable, and consistently implemented? | Were the outcome assessors blinded to the exposure status of participants? | Was loss to follow-up after baseline 20% or less? | Were key potential confounding variables measured and adjusted statistically for? |
| --- | --- | --- | --- | --- | --- | --- | --- | --- | --- | --- | --- | --- | --- | --- |
| Cata 2015 | Yes | Yes | Yes | Yes | No | Yes | Yes | Yes | Yes | NA | Yes | NA | Yes | Yes |
| Patino 2017 | Yes | Yes | Yes | Yes | No | No | Yes | Yes | Yes | NA | Yes | NA | Yes | Yes |
| Pang 2017 | Yes | Yes | Yes | Yes | Yes | Yes | Yes | Yes | Yes | NA | Yes | No | Yes | Yes |
